# Development of protein hydrolysate as a cost-effective and robust biodispersant: Investigating its performance in dispersing of carbon black pigment in water-based paints

**DOI:** 10.1016/j.heliyon.2024.e40036

**Published:** 2024-11-01

**Authors:** Mahmoud Reza Sadeghi, Hamid Saeidian, Zohreh Mirjafary, Morteza Rouhani

**Affiliations:** a Department of Chemistry, Science and Research Branch, Islamic Azad University, Tehran, Iran; b Department of Science, Payame Noor University (PNU), PO Box: 19395-4697, Tehran, Iran

**Keywords:** Pigment, Carbon black, Dispersant, Protein hydrolysate, Tinting strength

## Abstract

Carbon black pigments hold significant importance as the primary representatives of black pigments in the industry today. The dispersibility of carbon black pigment (CB) in water is limited by the nonpolar and weakly hydrophilic characteristics of the pigment's surface. Therefore, there is a critical need to devise an economical and eco-friendly approach for creating a well-dispersed and stable suspension of carbon black in an aqueous medium. The primary focus of this study was to investigate the dispersion capabilities of protein hydrolysate (HP) derived from sheep wool on CB particles in a water-based pigment concentrate. The hydrolysis degree of protein source was determined by high-performance liquid chromatography and gel permeation chromatography. The dispersion performance was investigated by zeta potential, transmission electron microscopy, Fourier transform infrared spectroscopy, grindometer, spectrophotometer-colorimeter, viscometer, and cryptometer measurements. The HP solution containing amino acids, peptides, and polypeptides with low molecular weight can cover the surface of the CB particles, creating enough electrical repulsion and steric resistance. As a result, this phenomenon can inhibit the collision and interaction among the pigment particles caused by Brownian motion, making it less prone to aggregation. The protein hydrolysate demonstrated a higher capability in producing the stable CB dispersions compared to a commercial reference dispersant, highlighting the effectiveness of amino acids, peptides, and polypeptides as powerful CB dispersing agents.

## Graphical abstract

**Figure:**
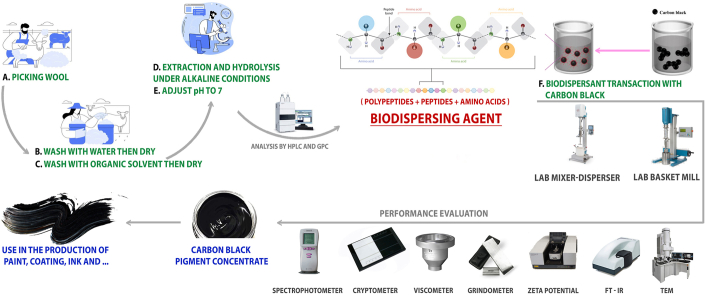

## 1 Introduction

Carbon black pigment (CB) stands out as a highly significant and extensively utilized pigment due to its exceptional opacity, remarkable tinting and painting capabilities. Consequently, it has emerged as the preferred option for CB in various applications [1]. CB particles exhibit both spherical and irregular crystalline structures of varying sizes, with the smallest dispersible units measuring between 50 and 500 nm. These unique characteristics, along with intense black color, make CB particles highly sought after in industries such as plastics, automotive manufacturing, and paint and ink production [2]. The exceptional tinting and coating capabilities of CB stem from its ability to absorb nearly all incident light and its fine particle size. CB pigments consist of extremely small primary particles with the tendency to form aggregates among each other, which in turn consist of chains and clusters.

To achieve optimal coating and a seamless film, the pigment particles, initially clumped together, must be dispersed and evenly distributed throughout the medium. A thorough distribution process results in a more uniform film appearance. A significant characteristic of CB lies in the presence of strong van der Waals interaction at the particle surface, facilitating accumulation and agglomeration, resulting in the formation of a carbon black mass. This impedes the dispersion of pigments within polymer resins of coatings, leading to a decline in the performance of CB. This issue is currently recognized as a major challenge in the paint and ink industries, as dispersing particles across various mediums proves to be energy-intensive, time-consuming, and costly. Consequently, agglomeration hampers CB dispersion in aqueous media, thereby restricting their application in water-based paints. Hence, enhancing the dispersibility of CB particles within water-based pigment concentrates would greatly broaden their utilization in the painting and coating industry. The thorough dispersion of pigment particles in the water-based paints significantly influences the color quality, leading to an increase in the tinting strength of color.

A method commonly used to disperse CB particles in the water-based pigment concentrates involves the addition of dispersing agents or the modification of the CB particle surface using large molecules or polymers [3,4]. These methods work by either creating electrostatic repulsion or steric hindrance between the CB particles, effectively keeping them apart or preventing their agglomeration and sedimentation. Previous research has demonstrated that CB particles can be dispersed in the aqueous phase through the adsorption of surfactants onto its surface. The colloidal stability of these surfactants is largely influenced by the quantity of surfactant adsorbed and the thickness of the resulting layer.

Establishing a stable dispersion and milling the CB using milling techniques becomes a complex task. Without the use of an appropriate dispersant or optimal concentration, the outcome often involves diminished tinting strength, necessitating larger quantities of concentrate for coloring purposes. Moreover, heightened viscosity levels can present obstacles during the dyeing process. To address these issues, the addition of polymers becomes essential to facilitate a uniform and suitable dispersion of particles in the aqueous phase. This intervention serves to reduce viscosity levels while enhancing the durability of the paint film. By incorporating polymers, the dispersion process becomes more efficient, leading to improved outcomes in terms of tinting strength and viscosity control [5].

In 2018, Kim et al. conducted a study focusing on the grafting of acrylic acid onto CB surfaces. However, the modified CB faced challenges in achieving stable dispersion in an aqueous solution due to a low grafting ratio. Acrylic acid and sodium allyl sulfonate were identified as effective chemicals for water absorption. The sulfonic group present in sodium allyl sulfonate is acknowledged for its strong electrolytic properties, which play a crucial role in enhancing the dispersion of CB particles in the water-based pigment concentrate. Consequently, the researchers opted to utilize a graft copolymer consisting of acrylic acid and sodium allyl sulfonate as modifying agents on the surface of CB particles. This strategic approach resulted in the successful achievement of excellent CB particle dispersion in the water-based paints [6]. Wang et al. employed the ene-thiol click reaction for the modification CB surface in a water-based pigment concentrate. They initiated the modification process by oxidizing the surface of the particles using various oxidizers, thereby generating hydroxyl and carboxyl functional groups on the surface of the CB particles. The oxidized CB particles were encapsulated by the sol-gel method with the γ-methacryloxypropyltrimethoxysilane, and then grafted with sodium 3-mercapto-1-propanesulfonate *via* thiol-ene click reaction. Modified CB particles show excellent self-dispersion ability in aqueous matrix and high thermal and centrifugal stability, even at 90 °C or 5000 r/min [7]. The modification of the CB particle surface using large molecules or polymers needs the multistep reactions which are requires special chemicals. It is evident that enhancing the dispersion of CB particles through surface modification requires a series of complex reactions and the utilization of specific chemical agents. This process involves multiple steps to achieve the desired outcome of improved dispersion.

Polymers that stabilize interfaces are commonly employed in various industries to maintain stable dispersions of colloidal particles in liquid solutions. While traditional dispersing agents based on poly(meth-)acrylate are suitable for use in organic solvents, there is a growing demand for the creation of eco-friendly water-based substitutes. A recent study introduced a new hydrophilic dispersing agent for CB particles that is based on partially hydrolyzed poly(*N*-vinylamide). These polymers have the ability to engage in hydrogen bonding and ion-dipole interactions, and can also be partially positively charged to facilitate ion pair formation, thereby enhancing their surface affinity. On the other hand changes in pH can have a profound impact on the interaction of the hydrophilic polymer and CB particles [8]. Fearon and colleagues conducted a study examining the impact of oxidized lignin (LigniOx) as a biodispersant in the dispersion of CB pigment. The research delved into the effects of various types of lignins on this process. The findings from measurements of viscosity, particle size, and zeta potential indicated that both hydrolyzed and non-hydrolyzed lignin samples were effective dispersants for CB pigment. Additionally, the study revealed that the LigniOx exhibited notable efficacy in dispersing TiO_2_ and CaCO_3_ pigments as well [9]. Various synthetic dispersants were additionally utilized for CB and various pigments in water-based pigment concentrates [[10], [11], [12], [13], [14], [15]].

**Fig. 1:**
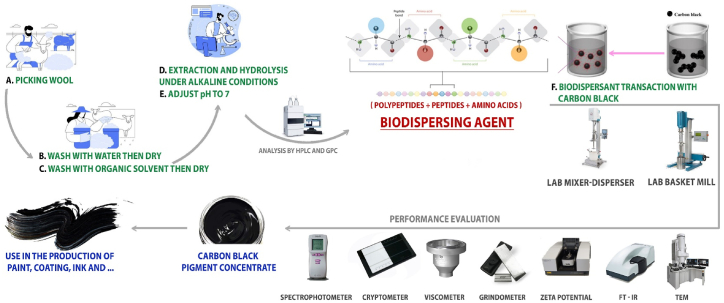
Overview of the current research.

The employment of biodispersants, such as amino acids, peptides, and polypeptides, for dispersing pigments in water, may be viewed as a beneficial measure in advancing environmental technologies. Hydrolyzed protein solutions (protein hydrolysate), characterized by their low molecular weight, find applications in various industries such as food, drilling, firefighting, animal feed, cosmetics and pharmaceuticals [[16], [17], [18], [19], [20]]. The protein hydrolysates serve as additives and ingredients in different products, showcasing their versatility and importance in multiple sectors. The protein hydrolysate solution may be derived from various plant sources, including soy, as well as animal sources, such as sheep wool and horns, *etc*. The general approach for generating a blend of amino acids, peptides, and polypeptides involves digestion of protein sources under enzymatic, acidic, or alkaline conditions [[21], [22], [23]]. The protein hydrolysate solution contains amino acids, peptides, and polypeptides, all of which possess carboxyl and amine polar groups. These groups have the capacity to transform into an ion pair depending on pH of the environment. The behavior of these compounds as dispersants closely resembles that of surfactants. Through spatial repulsion and electrostatic forces, they are capable of dispersing and stabilizing CB pigments in water, making them suitable for use as effective biodispersants in water-based pigment concentrates. The present study showcases the dispersion efficacy of a unique protein hydrolysate solution on CB pigment in water-based paints. The assessment procedure encompasses various techniques such as gel permeation chromatography, high-performance liquid chromatography, zeta potential (DLS) measurements, transmission electron microscopy (TEM), Fourier transform infrared spectroscopy (FT-IR), grindometer, spectrophotometer-colorimeter, viscometer, and cryptometer tests. Each technique offers unique advantages and contributes to a comprehensive understanding of the sample's behavior and attributes (Fig. 1). The performance of a reference dispersants (BASF 6225) in CB suspension was also evaluated based on the mentioned methods.

## 2 Experimental

### 2.1 Materials and instruments

The chemicals and instruments used in this research are given in Table 1, Table 2, respectively.

**Table 1:** The Chemicals used in the study.

Chemical	Manufacturer	Purity	Chemical	Manufacturer	Purity
Carbon Black 330	Iran Carbon	99.9 %	TiO_2_	Lomon Billions 996	95 %
Dispersing additive 6225	BASF	active ingredient 99.6 %	Viscofire PUR 60	BASF	active ingredient 40 %
anti-mold Biocide Silon K900	TaraChem	active ingredient 1.5 %	Propylene glycol	Shell	99 %
Defoamer Silon 508	TaraChem	active ingredient 25 %	Coalescing agent TexaSilon	TaraChem	99 %
Diethylene glycol	Arak Petrochemical	99 %	CaCO_3_ 800 mesh	Omya	98 %
Styrene acrylic resins	BASF	50 % solid in water

**Table 2:** The instruments used in the study.

Instrument	Model	Instrument	Model
FT-IR	Perkin-Elmer	Grindometer	Zehntner
TEM	DME-95-50	CryptoMeter	Kohler
spectrophotometer-colorimeter	Gretagmacbeth XTH	Zeta potential analyzer	Horiba SZ100
Viscometer	Ford Cup DIN 4	Gel permeation chromatography	Shimadzu LC-20A

### 2.2 Materials

#### 2.2.1 General procedure for preparation of the protein hydrolysate solution (HP)

Sheep's wool has been a popular choice for the preparation of the protein hydrolysate solution (HP) due to its affordability and accessibility. The preferred method for alkaline hydrolysis of wool involves the use of lime (CaO) as the hydrolyzing agent. In our recent study [24], lime was employed to ensure a more controlled process, ultimately maximizing the yield of peptides and polypeptides. The process included washing 1 kg of sheep wool with water and organic solvent to eliminate lipids, followed by adding it to a 10-L reactor equipped with a mechanical stirrer. Subsequently, 5 L of distilled water containing 150 g of hydrated lime were introduced to the reactor, which was then heated under 45 psi pressure. The stirring speed was maintained at 15 rpm for a duration of 1 h. The resulting liquid was neutralized to pH = 7.0 using HCl, filtered, and finally evaporated to a specific gravity of 1.12, resulting in the production of the final product (HP). To avert any potential bacterial contamination of the HP solution, methylchloroisothiazolinone (anti-mold Biocide Silon K900) was employed as a preservative, anti-mold and antimicrobial agent.

#### 2.2.2 General procedure for preparation of water-based pigment concentrate samples

Three distinct water-based pigment concentrate samples were prepared for the purpose of performance evaluation studies. The first sample (001) consisted of CB particles with a concentration of 30.0 wt% suspended in deionized water, which included defoamer, diethylene glycol, and anti-mold, without the use of a dispersant. It is important to note that a concentration of 30.0 wt% of CB in water is considered the highest and most suitable for the preparation of a water-based pigment concentrate. In the second sample (002), reference dispersant (BASF 6225) with a concentration of 5.0 wt% was used as a biodispersant and dissolved in deionized water. Subsequently, CB (30.0 wt%) was added to the solution. In the third sample (003), a protein hydrolysate (HP) solution with a concentration of 5.0 wt% were dissolved in deionized water, followed by the addition of CB (30.0 wt%) to the solution. Each sample underwent individual mixing at 800 rpm for a period of 30 min to ensure complete dispersion of the CB particles and then it was ground in a basket mill machine for 5 h. All three samples contained silicone defoamer (0.6 wt%), diethylene glycol (1.0 wt%), and anti-mold biocide silon K900 (0.10 wt%).

## 3 Results and discussion

The protein hydrolysate (HP) solution was subjected to an initial examination to identify the composition and proportion of free amino acids (AA) *via* amino acid analyzer (high-performance liquid chromatography, HPLC). Following this, to ascertain the total amino acid content, the aforementioned solution underwent acid hydrolysis with HCl (6 N) for complete hydrolysis, after which the resulting crude was analyzed using amino acid analyzer (Table 3).

**Table 3:** Free and total amino acid in the prepared HP solution.

Amino acid	Free amino acid (mg AA/g sample)	Total amino acid (mg AA/g sample)
Aspartic acid	2.2	17.6
Glutamic acid	3.8	41.7
Serine	0.7	1.0
Glycine	9.4	17.7
Histidine	–	1.0
Arginine	–	1.3
Threonine	–	–
Alanine	8.8	20.9
Proline	2.5	13.2
Tyrosine	1.3	8.9
Valine	0.6	10.7
Methionine	–	1.6
Cysteine	–	–
Isoleucine	0.5	10.9
Leucine	1.5	19.9
Phenylalanine	0.8	9.8
Tryptophan	1.0	11.5
Lysine	2.8	4.9
Total amino acids (%) per 100 g of protein hydrolysate solution	3.59	19.26

**Fig. 2:**
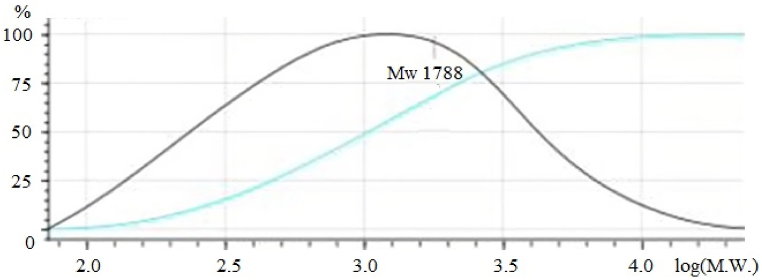
The molecular weight of amino acids, peptides, and polypeptides present in the protein hydrolysate solution.

As can be seen from Table 3, the amount of free and total amino acid in the HP solution is equal to 3.59 and 19.26 wt%, respectively. The highest amounts of amino acid in 1 g of the HP solution are glutamic acid (41.7 mg), alanine (20.9 mg) and leucine (19.9 mg). Gel permeation chromatography (GPC) was employed to analyze the molecular weight of amino acids, peptides, and polypeptides present in the HP solution (Fig. 2). The molecular weights of amino acids, peptides, and polypeptides in the HP solution range from 100 to 16000 Da, as illustrated in Fig. 2. The average molecular weight (Mw) of the components is 1788 Da.

The distribution of CB particles in the pigment concentrate was examined in accordance with the ASTM D1316 standard method [25]. The distribution of pigment particles in the CB concentrate was assessed by measuring the particle size of all three samples using a Grindometer device with a measurement range of 0–100 μm, and the results were recorded. It is important to note that samples 002 and 003 were subjected to three additional measurements after 1 h, 24 h, and 7 days to enhance precision. Interestingly, the initial observations, along with the repeated tests, did not show any significant changes in granulation. Both samples 002 and 003 had 10 μm granulation without scattered particles, while sample 001 had coarser granulation with scattering.

**Fig. 3:**
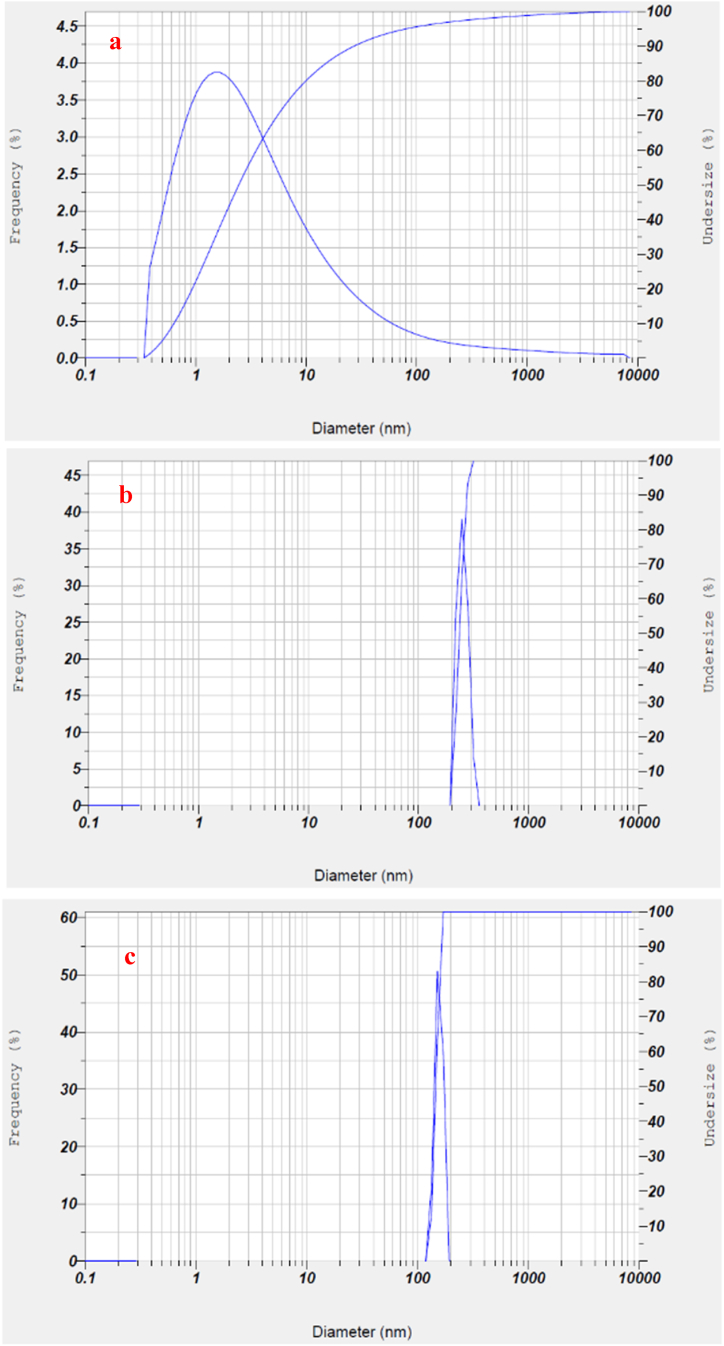
Size distributions of the CB particles in the water-based pigment concentrate 001 (a), 002 (b) and 003 (c).

Dynamic light scattering (DLS) analysis serves as a valuable method for determining the size distribution and stability of particles. The average hydrodynamic diameter of CB particles in samples 001, 002 and 003 was calculated to be 541.4, 319.2 and 162.6 nm, respectively. As can be seen from Fig. 3a, agglomeration of CB particles without a dispersant causes the formation of a broad size distribution peak. The results suggest that the addition of a dispersant in the water-based CB concentrates (Fig. 3b and c) and 003) results in a decrease in size and a narrower distribution of CB particles in the suspension. HP solution, functioning as a biodispersant, exhibits better efficacy than the reference dispersant in terms of the size and uniform dispersion of CB particles (Fig. 3c).

The zeta potential of −38.7 mV was observed in the CB suspension without a dispersant. Upon the addition of the reference dispersant, the absolute value of the zeta potential decreased to −30.5 mV. Subsequently, when the protein hydrolysate solution was introduced, there was a significant increase in the absolute value of the zeta potential, reaching −58.2 mV. The relationship between the absolute zeta potential and the strength of repulsion is evident, indicating that a higher absolute zeta potential results in stronger repulsion and consequently leads to a more stable system. These findings align with the outcomes of our experimental investigations. The incorporation of protein hydrolysate into the CB suspension facilitates interactions between the amino groups present in amino acids, peptides, and polypeptides with the CB particles, leading to their dispersion. These compounds, characterized by carboxylate groups that carry a negative charge, enhance the negative charge density of the CB particles. Consequently, the zeta potential of the concentrate is elevated to −58 mV.

**Fig. 4:**
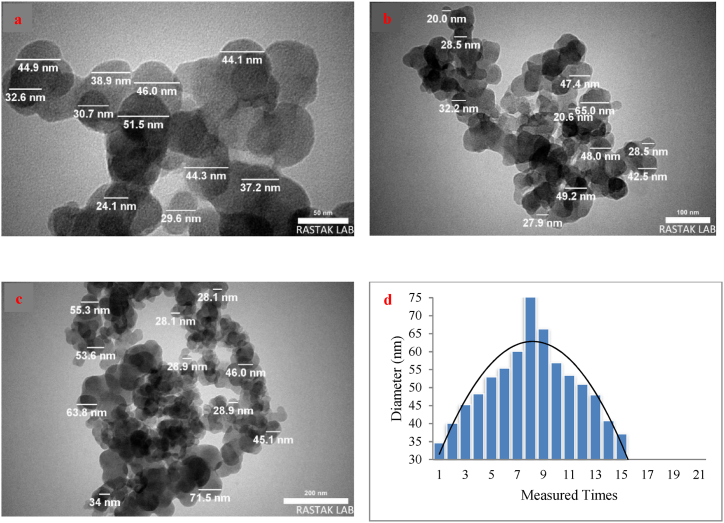
TEM images (a, b and c) and the particle size histogram (d) of the CB nanoparticles within the water-based pigment concentrate 003. The graph scale lengths are 50 (a), 100 (b) and 200 (c).

The transmission electron microscope (TEM) is a specialized instrument used to analyze the structure and morphology of nanoparticles at a high resolution. Through the examination and analysis of TEM images, valuable information regarding the material's structure can be obtained. Fig. 4a, b and 4c illustrates the TEM images and the particle size histogram of the CB nanoparticles within the water-based pigment concentrate 003, in the presence of HP as biodispersant. These images showcase the spherical nature of the CB nanoparticles and their size is less than 200 nm (Fig. 4d). Moreover, the TEM analysis confirms the lack of accumulation and agglomeration and highlights a consistent size distribution of the nanoparticles. TEM observations (Fig. 4) are in agreement with DLS results.

**Fig. 5:**
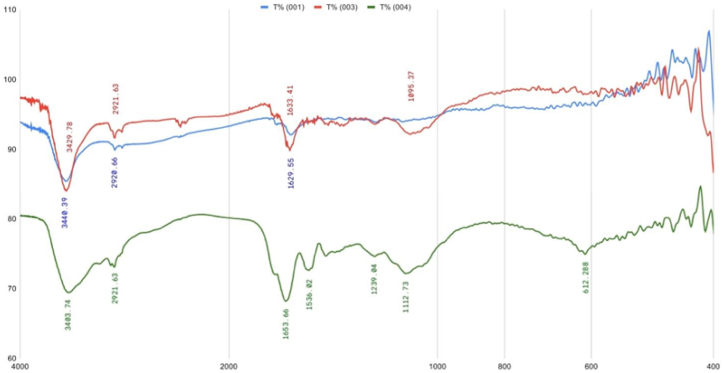
FT-IR spectral analysis of the water-based CB concentrate (001), the water-based CB concentrate containing the protein hydrolysate (003) and the solid of hydrolyzed protein solution (004).

FT-IR analyses were conducted on pigment concentrate 001 without a dispersant and 003 with the biodispersant HP to elucidate the interaction of amino acids, peptides, and polypeptides with CB particles. Additionally, FT-IR spectroscopy was utilized to analyze the vibrational frequencies of the solid of protein hydrolysate solution, which contained a variety of amino acids, peptides, and polypeptides with varying molecular weights (sample 004, Fig. 5). This approach facilitated a thorough comparison of the vibrational frequencies, revealing noticeable differences between the CB nanoparticles without a dispersant and those with protein hydrolysate as a dispersant.

The prominent band located at 3403.74 cm^−1^ in the FT-IR spectrum of sample 004 is associated with the stretching of -OH groups. The peak observed at 2921.63 cm^−1^ is attributed to the stretching of C-H bonds within the alkane moieties. Furthermore, the broad characteristic band seen at 1653.66 cm^−1^ signifies the vibration of the carbonyl (C=O) group. Moreover, the peak at 1112.73 cm^−1^ is indicative of the stretching of C-O bonds. Within the FT-IR spectrum of the CB suspension (001), the lack of a functional group results in the absence of any distinctive peaks, with the exception of the peak at 3440.39 cm^−1^, which is attributed to the hydroxyl group of water that has been absorbed onto the CB particle surface. The FT-IR analysis of the water-based CB concentrate (003) demonstrates the presence of frequencies linked to different vibrations in the HP, indicating the interaction between the HP constituents and the CB particles. Upon the adsorption of amino acids, peptides, and polypeptides onto the CB particles, noticeable changes were observed, such as the peak at 3403.74 shifting to 3429.78, 1653.66 to 1633.41, and 1112.73 to 1095.37 cm^−1^. The FT-IR results suggest that the components of the HP solution can effectively interact with the CB particles.

**Fig. 6:**
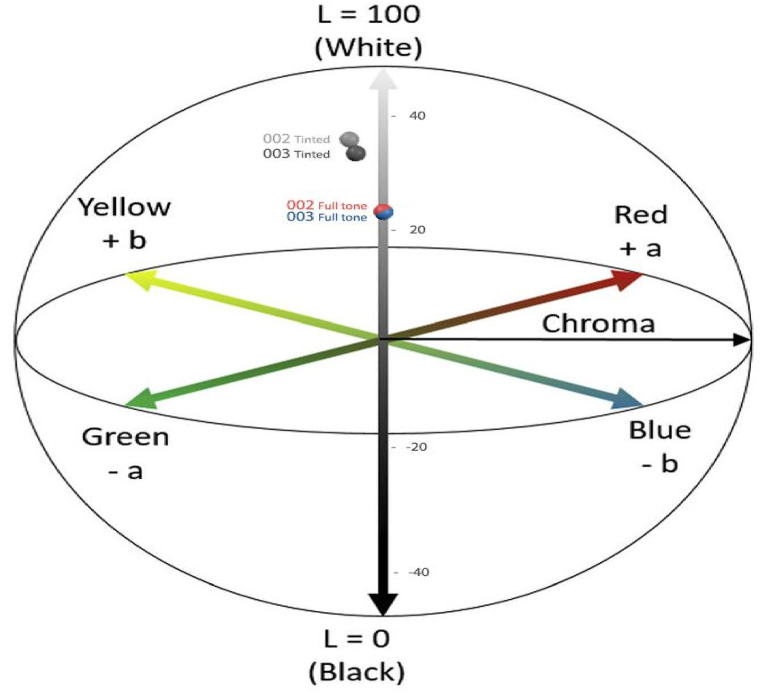
The CIELab color space of the prepared samples 002 and 003.

The spectrophotometer-colorimeter was employed to identify the darkness level and tinting strength of the prepared samples 002 and 003. The CIELab color space is widely recognized as the foundation of colorimetry [26]. This color space (Fig. 6) defines the values of (a) and (b) by utilizing four distinct colors within the human vision range, namely red, green, blue, and yellow. The values of (a) and (b) are defined in the range of −128 to +127. The variable (L) denotes the brightness on the vertical axis, ranging from 0 to 100, where 100 signifies the maximum brightness and 0 signifies the minimum brightness as well as the highest level of darkness. The comparison of the darkness and tinting strength of the prepared samples (002 and 003) can be accurately conducted through the utilization of the spectrophotometer-colorimeter in conjunction with CIELab results.

**Fig. 7:**
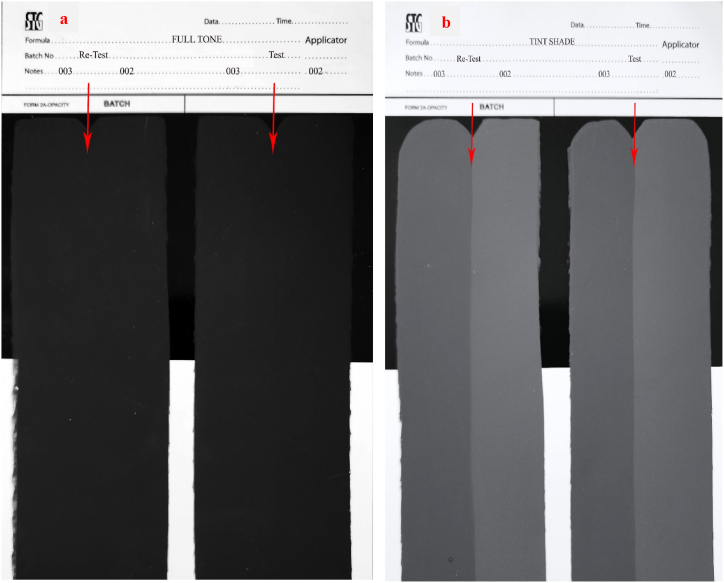
Lenta paper of the samples 002 (a) and 003 (b) for darkness and tinting tests.

In order to measure with a spectrophotometer-colorimeter, the initial step involves applying a film onto Lenta paper with a thickness of 100 μm. It is important to note that in order to assess the darkness, a combination of 8.0 wt% CB pigment concentrate (samples 002 and 003) and 25.0 wt% acrylic resin emulsion should be utilized. In order to assess the tint strength, a combination of water-based CB concentrates was employed alongside a white coating containing 25.0 wt% titanium pigment (TiO_2_), with a ratio of 2:98. The resulted mixture should be mixed for 5 min before being applied to create a film. Subsequently, the film is left to dry for 4 h at room temperature before being subjected to measurement and analysis (Fig. 7a and b).

The values of (a), (b) and (L) in darkness test for samples 002 and 003 are collected in Table 4. The findings from the colorimetric assessments (Fig. 7) and Table 4 demonstrate the ideal darkness of sample 003. A lower L value suggests a more preferable blackness in comparison to the reference sample 002.

**Table 4:** The values of (a), (b) and (L) in darkness test for samples 002 and 003.

Sample	(L)	(a)	(b)
002	24.06	0.12	0.11
003	23.96	0.90	0.16

The findings from the colorimetric analysis depicted in Fig. 7 and the data presented in Table 5 clearly indicate that sample 003 exhibits a tinting strength in comparison to sample 002. The color's tinting strength is influenced by various factors such as the type of pigment, including its natural transparency and chroma, as well as the quantity of pigment and the fineness of the grinding process. A finer grinding of the pigment results in a higher tinting strength, which in turn affects the paint's ability to retain its color when mixed with white color [27]. The existence of amino acid, peptide, and polypeptide in water-based CB concentrate 003 prevents the agglomeration of CB particles and promotes their uniform dispersion in the suspension. Consequently, this leads to a higher tinting strength of sample 003 in comparison to reference sample 002.

**Table 5:** The values of (a), (b) and (L) in tinting test for samples 002 and 003.

Sample	(L)	(a)	(b)
002	35.76	−0.82	−4.11
003	33.39	−0.62	−3.30

In order to assess the hiding power, a mixture of the water-based concentrate and acrylic resin emulsion was blended at a ratio of 10–90 for a duration of 5 min. Subsequently, the hiding power of the concentrate was evaluated using the CryptoMeter instrument and the ASTM D344 standard method [28], resulting in values of 38 and 40 m^2^/L for samples 002 and 003, respectively.

Typically, a decrease in viscosity of the pigment concentrate, contingent upon its stability, signifies the CB particles prevention from flocculation. Furthermore, lowering the viscosity through the using of dispersant additives, while ensuring performance is upheld, allows for the potential incorporation of larger quantities of pigment into the pigment concentrate composition. The viscosity of pigment concentrates 002 and 003 was determined using a Ford Cup DIN4 viscometer and the DIN 53211 standard method [29] at room temperature. It should be noted that without a dispersant, it was not possible to add 30.0 wt% of CB and control its granulation even to half of it. Even with a smaller amount of CB particles, it is involved in severe agglomeration and flocculation and lack of proportional distribution in the water-based pigment concentrate. The viscosity data indicates that sample 002 containing the reference dispersant, with a viscosity of 40 s, showed a thixotropic behavior after 24 h, and reverts to its original state upon re-mixing after 24 h. However, sample 003, with a viscosity of 40 s containing the HP biodispersant, remained stable after 24 h.

**Fig. 8:**
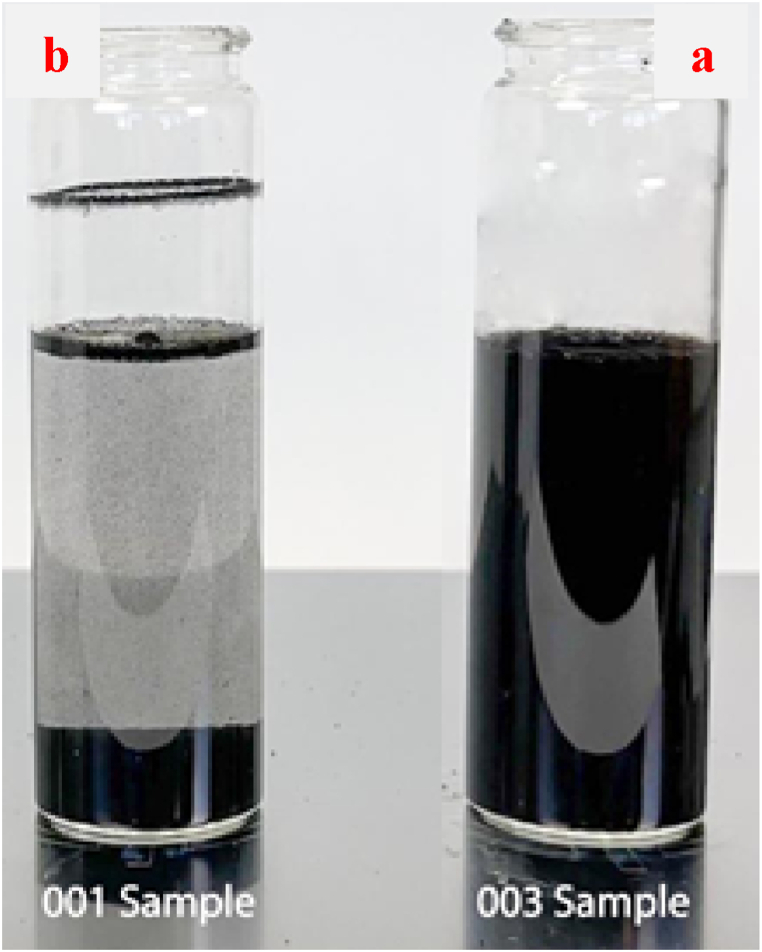
High stability of the water-based CB concentrate in the presence of the protein hydrolysate solution as an effective biodispersant (a) and without a dispersant (b).

Quality stability in the warehouse of the samples 001, 002 and 003 ensured by following the ASTM D2698-05 standard method [30]. The samples were centrifuged for 10 min at a speed of 5000 rpm and the results were observed qualitatively. The dispersant-free CB suspension demonstrates phase separation, whereas the concentrates containing a dispersant exhibit a stable suspension with no precipitation. Dispersion of CB prepared with the HP biodispersant (sample 003) and without a dispersant (sample 001) are illustrated in Fig. 8a and b, respectively.

A different stability study was also carried out as: both samples 002 and 003 were placed in an incubator at 40 °C for a week, followed by storage at 5 °C for another week. Throughout both stages, thorough observations were made, revealing no notable alterations in viscosity, granulation, or tinting strength. The samples exhibited complete uniformity, showing no signs of phase separation, soft or hard precipitation.

## 4 Conclusions

The dispersion characteristics of protein hydrolysate solution derived from sheep wool on carbon black pigment in aqueous system were first investigated in this study. The characteristics of carbon black dispersed with protein hydrolysate were analyzed through various methods including zeta potential, transmission electron microscopy, Fourier transform infrared spectroscopy, grindometer, spectrophotometer-colorimeter, viscometer, and cryptometer tests. Upon the addition of the biodispersant, the absolute value of the zeta potential increased to −58.2 mV. TEM images show the spherical nature of the CB particles and their size is less than 200 nm. The FT-IR results reveal that the components of the biodispersant solution can effectively interact with the CB particles. The findings indicated that the amino acids, peptides and polypeptides in protein hydrolysate solution were able to attach to the surface of the carbon black pigment, leading to a reduction in pigment size and an enhancement in the stability of the carbon black suspension.

In addition to the technical viability of the newly developed CB dispersant, several factors, including performance, environmental sustainability, cost-effectiveness, and availability, significantly influence the suitability of these dispersants for use in water-based paints. The process of preparing a hydrolyzed protein solution from sheep wool is economically advantageous and can be conducted under mild conditions. This hydrolyzed protein solution contains amino acids, peptides, and polypeptides, which are prevalent in both human and animal nutrition. The incorporation of these additives does not pose significant environmental risks, thereby rendering it a critical aspect in the evaluation of dispersing agents.

## CRediT authorship contribution statement

**Mahmoud Reza Sadeghi:** Writing – original draft, Methodology, Investigation, Formal analysis, Data curation, Conceptualization. **Hamid Saeidian:** Writing – review & editing, Supervision, Methodology, Investigation, Formal analysis, Data curation, Conceptualization. **Zohreh Mirjafary:** Writing – original draft, Formal analysis, Data curation. **Morteza Rouhani:** Writing – original draft, Formal analysis, Data curation.

## Data availability statement

Data will be made available on request.

## Declaration of competing interest

The authors declare that they have no known competing financial interests or personal relationships that could have appeared to influence the work reported in this paper.
